# Which Systolic Blood Pressure Measure Is Most Important for Determining Cardiovascular Risk: Seated or Supine Blood Pressure?

**DOI:** 10.1007/s11906-025-01346-3

**Published:** 2025-10-21

**Authors:** Tomas L. Bothe, Abigail E. Melloy, Andreas Patzak, Niklas Pilz

**Affiliations:** 1 Charité – Universitätsmedizin Berlin, Institute of Physiology, Center for Space Medicine and Extreme Environments Berlin, Chariteplatz 1, Berlin, 10117 Germany; 2 Charité – Universitätsmedizin Berlin, Institute of Translational Physiology, Berlin, Germany; 3 Hannover Medical School, Department of Cardiology and Angiology, Hannover, Germany

**Keywords:** Blood Pressure, Blood Pressure Measurement, Cardiovascular Risk, Supine Blood Pressure, Seated Blood Pressure, Hypertension Guideline

## Abstract

**Purpose of Review:**

In clinical practice, the diagnosis of hypertension is based on non-invasive upper-arm cuff blood pressure (BP) measurement. Most measurements are performed seated, although evidence indicates that supine BP may provide additional information. This review summarises recent findings on the influence of body posture on BP readings and cardiovascular (CV) risk prediction across office, ambulatory, and home BP monitoring (OBPM, ABPM, HBPM), their clinical implications and future research directions.

**Recent Findings:**

In OBPM, patients with supine-only hypertension demonstrated CV risk comparable to patients with hypertensive BP in both positions, and a higher risk than seated-only hypertensives. Supine hypertension was particularly predictive in individuals under 65 years of age. In ABPM, the strongest predictors of CV events are nocturnal hypertension and abnormal dipping patterns, particularly when patients are truly asleep, whereas supine nocturnal HBPM has been less extensively investigated.

**Summary:**

Current clinical practice remains primarily based on seated BP measurements. Recent trials have highlighted that supine OBPM may provide additional predictive power in the assessment of CV risk. These findings offer a partial explanation for the residual high predictive value of nocturnal BP values which can be derived from ABPM or specialised HBPM devices that goes beyond the correlation of breathing related sleep disorders Research should focus on homogenising supine risk data into composite risk scores combining seated and supine BP while new outcome studies should consider including supine BP measurement. Future guideline committees should consider recommending the structured clinical application of supine BP, given its demonstrated prognostic benefits.

**Graphical Abstract:**

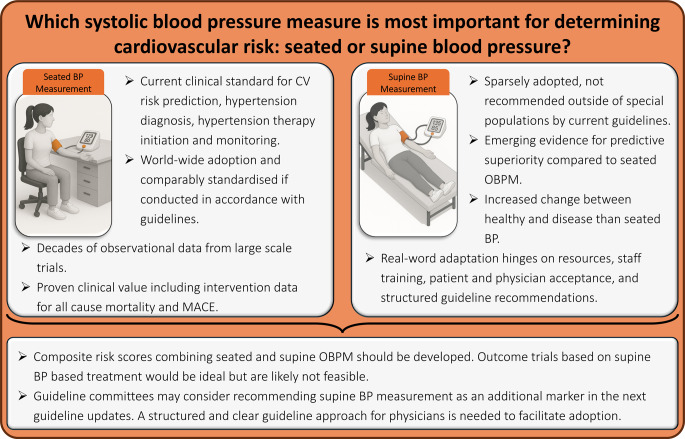

## Introduction

Arterial hypertension is a major risk factor for adverse cardiovascular (CV) events, including heart failure, coronary artery disease, heart attack, and stroke [1]. Non-invasive blood pressure (BP) measurement is predominantly performed using an upper arm cuff [2]. BP measurements can be conducted either as single assessments in hospitals or outpatient clinics by medical professionals (office BP measurement, OBPM), as long-term readings over a period of 24 h (ambulatory BP monitoring, ABPM) or at patients´ homes (home BP measurement, HBPM) [1]. Among these approaches, 24-hour ABPM is regarded as the gold standard for CV risk prediction [1, 3]. 

Since the early development of non-invasive BP assessments, Riva-Rocci recommended measuring BP in the seated position to minimise hydrostatic effects and to standardise the measurement conditions [4]. However, nocturnal dipping has shown the highest value in predicting adverse CV events across different BP parameters [5, 6]. While being clearly associated with breathing related sleep disorders (hazard ratios >3 for even moderate obstructive sleep apnoea), there is substantial residual, independent predictive value of nocturnal BP and dipping [7, 8]. Nocturnal dipping assessment relies on ABPM taken during the night, ideally while patients are fully supine in bed and confirmed to be asleep, rather than measured during “narrow-fixed intervals” (e.g. 22:00–06:00 h) [9, 10]. The change from dipping to non-dipping or reverse dipping patterns is introduced by a stronger increase in nocturnal BP compared to daytime BP with increasing CV disease burden, itself with multifactorial causes [11]. 

Emerging trial evidence has begun to question the long-standing conviction regarding the singularity and clear superiority of seated BP measurement [12–14]. This review synthesises recent literature comparing seated and supine BP in predicting CV events across OBPM, ABPM, and HBPM. We highlight recent key studies and discuss their clinical implications, as well as directions for future research.

### Current Guideline Recommendations

The leading international hypertension societies, including the International Society of Hypertension (ISH), the European Society of Hypertension (ESH), the European Society of Cardiology (ESC), the American Heart Association (AHA), and the American College of Cardiology (ACC), recommend ABPM as the confirmatory tool for diagnosing arterial hypertension [1, 15, 16]. If ABPM is not feasible or available, HBPM should be performed. As the primary initial assessment, OBPM is widely applied in everyday clinical practice. OBPM should be conducted with the patient at full rest, seated, with feet flat on the floor, back and arm supported, and cuff positioned at heart level. Neither the ESH nor the AHA/ACC guidelines recommend supine BP measurements for routine OBPM or HBPM, with the latest 2025 AHA/ACC guidelines mentioning supine BP only in the context of diagnosing orthostatic hypotension [16]. While treatment initiation thresholds and targets are based on both systolic and diastolic BP, systolic BP has emerged as the primary predictor of CV risk. This is reflected in guideline recommended CV risk scores such as the European Society of Cardiology’s Heart Score (SCORE 2) [1, 17]. 

### Physiological Considerations on Body posture, Hydrostatic Pressure and BP Measurement

Unless counteracted by autonomic regulation, Earth’s gravity induces a fluid shift towards the lower body in upright positions, thereby reducing venous return and stroke volume. This physical effect leads to differential effects in patient groups in which primarily healthy subjects show a reduction in BP when being supine while patients with preexisting disease tend to show an increase in both systolic and diastolic supine BP compared to seated measurements [18–21]. Supine BP, such as nighttime BP readings, may therefore more closely align with physiological transitions from a healthy to a diseased state and could therefore provide greater prognostic value [22, 23]. 

During nocturnal ABPM, changes in body posture alter the hydrostatic pressure difference between heart level and the cuff. A recent study reported that postural changes during sleep led to average underestimations of systolic BP by nearly 10 mmHg when the cuff was positioned above heart level. After correcting for the hydrostatic pressure difference between the cuff and heart level, 27.5% of patients changed their nocturnal hypertension classification while 37.3% of patients changed their nocturnal dipping pattern. A total of 49.0% of patients were affected by either one or both changes. All but one patient transitioned towards a less favourable hypertension and/or dipping pattern after correction [24]. 

### Reason for Conducting Seated BP Measurement

In both research and everyday clinical practice, most BP assessments are conducted in the seated position. The main advantages are its simplicity, familiarity for physicians and patients, minimal equipment requirements, and potential for standardisation [1]. Seated OBPM permits rapid assessment with virtually no exclusion criteria. In theory, its familiarity and ease of application should result in a high reproducibility of seated BP measurements. Owing to its proven clinical value, practicality, and broad applicability, seated OBPM has remained the reference standard for most clinical outcome studies and for BP measurement device validation protocols [25, 26]. 

Most importantly, all current treatment guidelines, including BP treatment targets and intervention thresholds, are based on seated OBPM measurements. At present, patient diagnosis and management are primarily based on this traditional measurement approach [1, 15, 16]. 

### Current Evidence Comparing Seated and Supine BP

#### OBPM

In everyday clinical practice, OBPM is performed almost exclusively in the seated position. However, recent large-scale studies have incorporated additional supine measurements, enabling direct comparisons between the two postures.

The ARIC study assessed both seated and supine OBPM in more than 11,000 participants across 26 years of follow-up. Supine hypertension was identified in 16.4% of patients without seated hypertension and in 73.5% of those with seated hypertension. Participants with supine hypertension had a higher risk of all-cause mortality, heart failure, coronary heart disease, and stroke compared with those with normal supine BP. Notably, patients with hypertension confined to the supine position had a CV risk comparable to those assessed as hypertensive in both seated and supine positions, and a higher risk than those classified as hypertensive only when seated [13]. This suggests that the improved CV risk prediction associated with supine BP is more complex than simply reflecting higher average BP values.

The KoGES study assessed OBPM sequentially in the seated, supine, and standing positions in more than 8,000 participants over 10 years of follow-up. Supine systolic hypertension emerged as the strongest predictor of all-cause mortality. In comparison, seated BP was higher than supine BP for both systolic (seated: 119.6 mmHg; supine: 115.7 mmHg, difference: 3.9 mmHg) and diastolic (seated: 79.4 mmHg; supine: 74.3 mmHg, difference: 5.1 mmHg) BP. Among patients younger than 65 years, only supine hypertension predicted CV mortality, whereas in those aged ≥ 65 years, hypertension in any posture was predictive, with supine BP remaining the most predictive measure [21]. Similar findings, with supine BP proving superior in predicting major adverse CV events (MACE) were reported in a large Chinese cohort of elderly patients [14]. Classical Norwegian follow-up mortality data further support this observation [27]. 

Beyond MACE and overall mortality, supine OBPM has also been associated with subclinical target organ damage. In a cohort of 165 hypertensive patients, unattended supine systolic BP demonstrated the strongest correlation with markers of hypertensive organ damage, including arterial stiffness and reduced renal function, outperforming even ABPM in predicting a composite organ damage score [28]. These findings are supported by a cohort of 590 patients in which supine OBPM was more predictive than seated values recorded in an OBPM setting for left ventricular hypertrophy and the urine albumin-creatinine ratio [29]. 

#### ABPM

ABPM provides repeated BP readings over a 24-hour period, capturing values during patients’ daily activities as well as during sleep. Accordingly, BP values in different postures are recorded, with upright measurements predominating during the daytime and supine measurements during the night [24]. In addition to postural influences, physical activity also affects ABPM, with daytime BP tending to be higher after standing and movement compared with periods of being seated [30]. This most strongly affects systolic BP but is also detectable in mean and diastolic BP values [31]. The prognostic relevance of daytime versus nighttime ABPM has been demonstrated across diverse populations [5]. Multiple investigations have reported strong associations of elevated nighttime BP and impaired or even reversed nocturnal dipping profiles with CV outcomes and hypertension-mediated organ damages [32–34]. A relevant proportion of changes in nocturnal BP can be explained by the high co-occurrence of non-dipping patterns in BP and breathing related sleep disorders which themselves are highly predictive of MACE [7, 8]. The stronger increase of nocturnal BP in comparison to daytime could explain the predictive advantage of nocturnal BP [11]. A similar trend could be observed for the comparison of seated and supine BP [18–21]. 

However, the reproducibility of nocturnal ABPM indices is limited, which poses challenges for serial risk stratification [35, 36]. One study reported that conventional ABPM may underestimate true nighttime BP because the cuff often remains above heart level during sleep, resulting in hydrostatic pressure differences between the heart and the cuff. After correcting for offsets between cuff and heart level, nearly half of the participants were reclassified regarding their dipping pattern or nocturnal hypertension status. Furthermore, substantial differences between possible nocturnal body positions including supine, left side, right side, prone were observed, causing additional uncertainty when interpreting nocturnal BP data [24]. This highlights the need for technological advancements, such as wearable position sensors, to enable automatic correction of body position changes, as well as further analyses into the body-position-specific risk prediction potential of nocturnal BP. Future research will need to employ advanced technologies to further understand the difference between sleep related causes (e.g., obstructive sleep apnoea) and changes in nocturnal BP, ideally while not affecting the patients’ sleep with repeated cuff inflations [5, 37, 38]. 

#### HBPM

While HBPM offers clear benefits in patient comfort and empowerment, its reproducibility is substantially limited, primarily due to flawed execution of BP measurement outside guideline recommendations [39, 40]. Compared to sporadic office readings, HBPM provides superior prediction of stroke risk [41, 42]. As patients are generally instructed to sit during HBPM, the influence of body posture on HBPM has been less thoroughly investigated [43]. 

Technological advances have enabled automatic HBPM devices capable of capturing sparse nocturnal readings while patients are asleep. The J-HOP study compared the prognostic value of nocturnal HBPM and ABPM in more than 3,000 patients. Nocturnal hypertension assessed by HBPM was independently associated with increased MACE risk, irrespective of OBPM levels [44]. Importantly, nocturnal HBPM may reduce the misdiagnosis of masked hypertension and may be more acceptable to patients owing to fewer cuff inflations compared to ABPM. Thus, supine HBPM, though less extensively studied, appears to provide additional prognostic information.

### Clinical Interpretation and Implications for Practice

Epidemiological data suggest that isolated supine hypertension is a relatively frequent phenomenon, with affected patients experiencing relevant increases in MACE event rates [13]. Incorporating supine BP into the diagnostic work-up may enhance risk stratification beyond standard seated OBPM. The predictive superiority of supine BP in individuals younger than 65 years, as suggested by the KoGES cohort, indicates that early stratification based on supine BP could help identify younger patients requiring timely intervention to prevent long-term CV events [13]. 

Despite compelling evidence, measuring BP in multiple positions is time-consuming and therefore difficult to integrate into routine clinical practice. This is particularly relevant given that the majority of BP measurements, especially OBPM and HBPM, are not performed in accordance with guideline recommendations or are substantially affected by measurement artefacts, as is frequently the case with ABPM [31, 39, 45, 46]. It is important to note that the predictive advantage of supine OBPM measurement may not be replicable in more “opaque” real-world measurement scenarios. Potential sources of artefacts, such as patients not lying fully flat (e.g. bent legs) or maintaining suboptimal arm positions, may diminish the predictive advantage of supine BP measurement.

Pragmatically, supine BP assessment may be prioritised in specific scenarios, such as in patients with high-risk profiles or with inconclusive seated BP results near diagnostic thresholds. Supine BP measurement is also relevant when ABPM cannot be performed or tolerated. However, there are no clearly standardised guidelines for conducting supine BP measurement, nor are there clearly defined supine BP treatment threshold or targets available. Considering e emerging evidence and the need for clear, standardised approaches to support effective clinical implementation, guideline committees should revisit incorporating a structured approach to supine BP measurement and interpretation into future recommendations.

## Recommendations for Future Research

Most evidence on supine BP is derived from observational studies, inherently limiting causal inference. It remains uncertain whether lowering isolated supine hypertension in patients with normal seated BP translates into improved clinical outcomes.

Furthermore, it is unclear whether lowering supine BP is an independent predictor of reduced MACE risk, beyond the lowering of seated BP. As with HBPM and ABPM in general, the absence of randomised controlled treatment trials delivering hard outcome data, coupled with the lack of clearly defined treatment thresholds and target values, limits the potential replacement of seated OBPM with supine BP measurement in real-world practice. Whilst such data is urgently needed, conducting the requisite studies poses substantial methodological challenges and is unlikely to be feasible in practice? As an intermediate step, composite risk scores that integrate both seated and supine BP could improve CV risk stratification.

Technological innovation in BP devices with integrated posture sensors or hydrostatic compensation algorithms could improve measurement accuracy and reproducibility. Devices that automatically record body posture would enable posture-specific BP values and could enhance improved CV risk prediction as well as facilitate adjustment of BP readings [31, 46]. 

## Summary

Arterial hypertension remains the leading cause of CV morbidity and mortality worldwide. Accurate BP measurement is therefore fundamental for diagnosis and risk stratification. Nevertheless, current guidelines and clinical practice remain anchored almost exclusively in seated OBPM, with thresholds and treatment targets based solely on seated values. Supine measurements are mentioned in current international guidelines only in the context of diagnosing orthostatic hypotension, leaving a critical gap in routine CV risk assessment. Although seated OBPM is simple and well established in clinical outcome studies, it may limit the ability to optimally assess CV risk.

Emerging epidemiological evidence demonstrates that supine BP is a superior predictor of CV risk. The ARIC study found that patient’s hypertensive only in the supine position carried risks of death, and MACE comparable to those hypertensives in both positions, and substantially greater than those hypertensive only when seated. Likewise, the KoGES cohort identified supine systolic hypertension as the strongest predictor of all-cause mortality, most notably in individuals under 65 years, in whom seated BP alone failed to predict CV mortality. Additional large-scale studies corroborate that supine BP is more strongly linked to adverse CV outcomes and subclinical organ damage, including arterial stiffness, renal dysfunction, left ventricular hypertrophy, and microalbuminuria.

Supine BP may more closely reflect central haemodynamics than seated values. Further, is appears to track the transition from a healthy state towards a state with higher CV risk more clearly and with a greater change in BP when compared to seated BP, offering a potential explanation for its predictive advantage.

Clinically, supine BP assessment should be prioritised in high-risk patients or in cases with inconclusive seated readings near diagnostic thresholds, especially when ABPM is not feasible. Supine BP consistently reveals CV risk that seated readings fail to identify. Future approaches should rely on developing composite risk scores integrating both seated and supine BP, together with technological innovations such as posture-sensing or hydrostatically corrected devices (especially in ABPM). Given the exceptional potential of supine BP to improve CV care, guideline committees should recognise supine BP as an attainable and impactful component of CV risk assessment and begin to recommend its structured clinical application beyond special populations.

## Data Availability

No datasets were generated or analysed during the current study.
